# Dependence of Biocatalysis on D/H Ratio: Possible Fundamental Differences for High-Level Biological Taxons

**DOI:** 10.3390/molecules25184173

**Published:** 2020-09-11

**Authors:** Igor Zlatskiy, Tatiana Pleteneva, Alexander Skripnikov, Tatiana Grebennikova, Tatiana Maksimova, Nadine Antipova, Olga Levitskaya, Mariia Makarova, Igor Selivanenko, Anton Syroeshkin

**Affiliations:** 1 Peoples Friendship University of Russia (RUDN University), 6 Miklukho-Maklaya St, 117198 Moscow, Russia; tvplet@mail.ru (T.P.); t_grebennikova@mail.ru (T.G.); maximtat@mail.ru (T.M.); antipova.nadine@gmail.com (N.A.); levitskayavolha@gmail.com (O.L.); tabulett6@gmail.com (M.M.); livmatter@mail.ru (A.S.); 2 State Institute of Genetic and Regenerative Medicine NAMS of Ukraine, 04114 Kyiv, Ukraine; 3 Shemyakin-Ovchinnikov Institute of Bioorganic Chemistry of the Russian Academy of Sciences, 16/10 Miklukho-Maklaya, 199997 Moscow, Russia; a.skripnikov@gmail.com; 4 Federal Research Center of Epidemiology and Microbiology named after N.F. Gamaleya, 117198 Moscow, Russia; 5 Mendeleev University of Chemical Technology of Russia, 117198 Moscow, Russia; selivanenko@mail.ru

**Keywords:** isotopic composition of water, deuterium-depleted water, deuterated water, living organisms, kinetic model, biocatalytic scheme

## Abstract

The kinetics of biological reactions depends on the deuterium/protium (D/H) ratio in water. In this work, we describe the kinetic model of biocatalytic reactions in living organisms depending on the D/H ratio. We show that a change in the lifetime or other characteristics of the vital activity of some organisms in response to a decrease or increase in the content of deuterium in the environment can be a sign of a difference in taxons. For animals—this is a curve with saturation according to the Gauss’s principle, for plants—it is the Poisson dependence, for bacteria a weakly saturated curve with a slight reaction to the deuterium/protium ratio toward increasing deuterium. The biological activity of the aquatic environment with reduced, elevated, and natural concentrations of deuterium is considered. The results of the study are presented in different vital indicators of some taxons: the bacteria kingdom—the colony forming units (CFU) index (*Escherichia coli*); animals—the activation energy of the death of ciliates (*Spirostomum ambiguum*), embryogenesis of fish (*Brachydanio rerio*)*;* plants—germination and accumulation of trace elements *Callisia fragrans* L., sprouting of gametophores and peptidomics of moss *Physcomitrella patens*. It was found that many organisms change their metabolism and activity, responding to both high and low concentrations of deuterium in water.

## 1. Introduction

All living objects contain a large amount of water, which is the habitat for many of them. Nutrient (vital) substances enter the organism with water and metabolic products are removed by water [1,2]. The full absorption of nutrients as well as the complete removal of biotransformation products depend on the intensity and nature of the metabolism. In the process of evolution, animals and plant organisms developed the ability to self-regenerate and reproduce, which is based on the metabolism, which in turn, depends on the properties of water as a habitat or food source (dissolved minerals) [3,4]. Therefore, the task of improving water quality, besides the purification, was added with the idea of changing its physicochemical properties or quality, in order to optimize metabolism processes [5].

Natural water contains hydrogen isotopes (protium, deuterium, and tritium) in different proportions. Evolutionarily, all living organisms are adapted to natural water with a constant ratio of protium and deuterium [6,7]. Some physicochemical properties of water with different hydrogen isotope content presented in Table 1. Therefore, it can be assumed that a change in the isotopic composition of water leads to a modification of the vital indicators of eukaryotes and prokaryotes. Moreover, natural water contains 6400 times more protium than deuterium, so it is likely that living organisms better tolerate the deuterium-depleted water than the deuterated one [8].

It is proven that biological systems are the most sensitive to isotopic effects involving deuterium [9,10]. In organic chemical compounds that are part of living tissues, deuterated water is more stable and inactive. Therefore, it is not included in metabolic processes and inhibits them [11,12]. The presence of deuterium leads to a change in intracellular structures and properties of living systems [13,14,15]. The most important for the macromolecule are hydrogen (deuterium) bonds. They are formed between adjacent atoms of deuterium (hydrogen) and heteroatoms of oxygen, carbon, nitrogen, sulfur, etc. They play one of the main roles in determining the structures of macromolecular chains and intracellular processes. Using different biological and chemical substances as an example, it was studied that different kinetic isotope effects are observed in deuterium-depleted and deuterated waters [16]. This effect is proportional to the concentration of deuterium in the medium and is especially pronounced at lower concentrations compared to natural ones [17,18].

The information on the biological effects of water with a modified isotopic composition on living organisms of different taxonomic levels is insufficient. The research in this direction is incomplete. Therefore, the purpose of this work was to study the effect of D/H ratio in water on the vital indicators of some organisms belonging to different taxons, to identify sensitivity and general mechanisms of action.

## 2. Results and Discussion

### 2.1. General Characteristics of Changes in Indicators of Various Taxonomic Groups of Organisms in Water with Different D/H Isotopic Ratios

As a result of research, we determined the effect of different concentrations of deuterium in water on the change in the values of the indices of some groups of taxons. All the values were conventionally given in one system of result assay (in percentage terms, where the results obtained in water with natural isotopic composition were taken as 100%) to unify the presentation of changes in the values of different indicators (growth, survival, embryotoxicity, etc.) (Table 2).

In bacteria, according to the results of changes in the values of the colony forming units (CFU) of *E. coli*, we noted the inhibition of colony growth in an environment based on the deuterated water. Therefore, this representative of the bacteria kingdom can be evaluated as an indicator of changes in the D/H isotopic ratio in water toward enrichment with deuterium.

Changes in the moss peptidom under the conditions of sprouting in the deuterium-depleted water led to a 30% change compared to sprouting on water with the natural ratio of deuterium. We did not observe moss growth in the deuterated water, which makes the representative of this taxon highly sensitive to the deuterium content in water.

Changes in the accumulation of *C. fragrans* L. in waters with different D/H isotopic compositions show that the redistribution and assimilation of mineral components in the deuterium-depleted water goes much faster—two or more times faster than in water with the natural isotopic composition. The accumulation and germination on the deuterated water did not give statistically significant results. That is *C. fragrans* L. can be classified as a highly sensitive representative of the kingdom of higher plants with respect to changes in the deuterium content in water.

The survival values of the unicellular biosensor *S.ambiguum* show the dependence on the D/H isotopic ratio in water according to the principle of the distribution of the Gaussian curve, taking into account the previously presented data [17,19]. Optimal lifespan values correlated with the natural D/H ratio in water. It is interesting that in this case Ea does not change, as we noted it earlier [19].

When studying embryogenesis of fish, it is worth noting that in the deuterium-depleted environment, high mortality (a chronic level of toxicity) was observed, compared with the control (natural D/H ratio). The acute toxicity of 50% was observed in the deuterated medium. In the deuterated environment, sublethal signs of toxicity were also recorded—a slowdown (delay) in the fish embryogenesis. Therefore, the indicator of embryogenesis of fish can be classified as highly sensitive with respect to changes in the D/H ratio in water.

According to the obtained results [1,20,21], it is noticeable that the deuterated water significantly reduces the activity of all indicators in different groups of organisms. The analysis of changes in the vital indicators of the organisms in the deuterium-depleted water environment does not give a definite clear correlation. Some biological effects of water with a modified isotopic composition was presented earlier [1,17,19,22,23,24,25]. The results of deuterium-depleted water exposure on the lower and higher plants, not previously published considered below.

### 2.2. Change in CFU of E. Coli in a Medium with Different D/H Isotopic Ratios

As an example of the reaction of a bacterial culture to a change in the isotopic composition of the medium, the effect of the heavy water (D_2_O) on the *E. coli* culture was investigated. The physiological state of the cell changes with increasing or decreasing deuterium content in water [1]. We found a rearrangement of the size spectrum of the culture during the growth of *E. coli* in D_2_O: the number of single cells decreases sharply, the aggregation of the culture takes place with the formation of associates of 4, 10 and 70 μm in size. The numerical and volume distribution of cells and associates of *E. coli* culture also varies; in the control culture grown in the environment of the natural D/H isotopic ratio the volume concentration of biomass was 0.024% and in the culture grown in D_2_O—0.008%.

It is worth noting that with the growth of the *E. coli* culture in an environment based on the deuterium-depleted water, no statistically significant deviations of the results from the control culture were observed.

### 2.3. Change in the Accumulation of Minerals during the Germination of *C. fragrans* L. in Water with Different D/H Isotopic Ratios

The content of zinc in the leaves of *C. fragrans* L. after saturation with zinc glycinate increased to 1.5–2 μg/g. At the starting points, the content of zinc was 0.01–0.02 μg/g. Furthermore, we observed twofold variability of the initial content of zinc and other trace elements while maintaining the species profile (biogeochemical profile). It is interesting that during the incubation of shoots in the solution of zinc glycinate, zinc was unevenly accumulated in the lamina over the entire length of the lamina. The highest zinc saturation was noted in the lower third (in the petiole) for leaves of 8–10 cm. The control of the topology and the uniform distribution of zinc allowed the X-ray diffraction analysis with intravital focusing on various zones of the lamina.

Table 3 shows how the coefficient of zinc accumulation in the leaves of *C. fragrans* L. changes during the incubation of shoots in solutions of zinc glycinate (20 mM Gly, 1 mM Zn^2+^, pH 7.0) in water with the natural D/H ratio and deuterium-depleted water.

It should be noted that a change in the isotopic composition of water made it possible, as expected, to achieve zinc accumulation two orders of magnitude higher than the natural D/H ratio in water. It cannot but emphasizes that similar results of Zn enrichment were obtained for another species—*Kalanchoe daigremontianae* [26]. It is interesting that iron has a low dynamics of accumulation, while the manganese content (Table 3) either does not increase or (in ddw) decreases slightly. In our opinion, this change in the elemental profile indicates not only the accumulation of zinc, but also a change in the metabolism of macroelements in the deuterium-depleted water [27].

The increase in the zinc content in the plant compared to the biogeochemical norm of the species has natural limitations, which follows from the Nernst equation and is caused by the natural accumulating ability of the plant at the concentration of free dissolved chelating agents and sorption at the phase interface. An increase in the assimilation ability of a plant is possible due to a change in the isotopic composition of the aqueous solution used for irrigation or in hydroponics. Instead of ordinary aqueous solutions, we use water depleted in heavy isotopes, which differs from ordinary water in some physicochemical parameters, including the freezing point, proton spin-spin relaxation time, and self-diffusion coefficient [28,29]. The reaction of living systems to the depletion of an aqueous solution with respect to the deuterium content, oxygen isotopes 17 and 18 is usually described as a change in metabolism [30,31]. One of the most interesting consequences of this change in the isotopic composition of water is a change in the rate constants of absorption/release of the solute. This is the effect that allows us to “circumvent” the thermodynamic limitations in nonequilibrium conditions, increasing the saturation of the medicinal plant with zinc compounds.

Thus, the use of zinc glycinate solutions in the deuterium-depleted water altered the metabolism of C. fragrans. A 200-fold accumulation of zinc in the lower third of the lamina up to 2 mg per 1 g of dry weight was noted. This result will allow offering special metal-modified medicinal and spicy-aromatic plants to compensate for zinc-deficient conditions in the future.

We also noted the inhibition of *C. fragrans* growth in the deuterated water, which did not allow the elemental analysis.

### 2.4. Change in the P. patens Moss Peptidom When Sprouting in Water with Different D/H Isotope Ratios

Studies of changes in the peptide composition of living organisms are very promising and interesting from the point of view of identifying intracellular mechanisms in response to stimuli of any kind and order. The increased interest in the most ancient plant, *P. patens* moss, is associated with the fact that it has become a leading model in the study of molecular mechanisms of plant development [32,33,34]. The relatively large genome of *P. patens* was sequenced and became available in open databases for comparative genomic evolutionary studies almost immediately after determining the complete genomic sequences of rice and poplar as well as of three types of green algae [35]. *P. patens* moss, widely used as a model system for molecular biology and ontogenetic studies, has become an important fundamental object of proteomics. To date, two independent projects for proteomic mapping of *P. patens* moss were implemented in Germany [36] and Korea [37]. As a result, using the MASCOT search system and the BLASTX comparison software, more than 300 proteins isolated from the protonema were processed [36].

We studied the peptide composition of the *P. patens* moss under sprouting conditions on deuterium-depleted and natural isotopic composition media. It should be noted that we did not observe moss growth with the deuterated water, which makes the representative of this taxon highly sensitive to the increased deuterium content.

Table 4 presents a partial peptidom of moss sprouted on waters with the natural D/H isotopic ratio and deuterium-depleted. It should be noted that 1–47 peptide residues were noted only in the sprouted moss based on water with the natural D/H ratio and were not determined in the medium based on DDW. Peptide residues 48–86 were determined only in the peptidom of moss sprouted based on deuterium-depleted water, and were not defined in the comparison group based on water with the natural D/H isotopic ration.

Thus, it should be noted that a change in the D/H isotopic composition leads to a significant peptide change in the composition of moss, which can lead to changes in the metabolism of the entire organism.

### 2.5. Kinetic Model of the Vital Activity of Living Organisms Depending on the D/H Ratio

**Figure 1:**
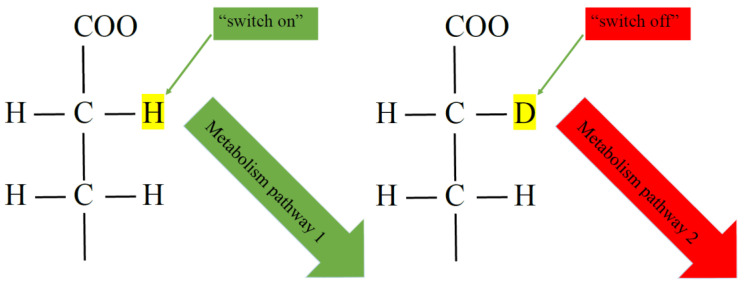
Scheme of changes in deuterium metabolism depending on chirality according to the “switch on/off” mechanism.

It was previously noted that with a lack of deuterium, the rate of metabolic reactions changes [38,39,40,41,42,43]. Moreover, from the point of view of the classical concepts of the dilution of substances, deuterium concentrations <100 ppm, at which changes in metabolism are observed, should be insignificant, but the observed effects are reliable. Therefore, deuterium acts as an element that is necessary as a regulator of the rate of reactions in complexes and cascades of biochemical reactions. On the other hand, deuterium can be considered to be an element that affects the chirality of the substance [17,44], which can explain the mechanism of many changes as a result of different D/H ratios (Figure 1). In other words, the presence of deuterium or protium in the substance (“switch on/off” mechanism) leads to different ways of further reactions and, respectively, the entire metabolism can go in different directions (at different rates) depending on the presence of deuterium or protium in the original or intermediate substance. If we accept this assumption, the key metabolic regions and reaction pathways, in which the deuterium/protium ratio can act as a trigger, as a regulator of metabolic processes according to the “switch on/off” system, remain unknown.

**Figure 2:**
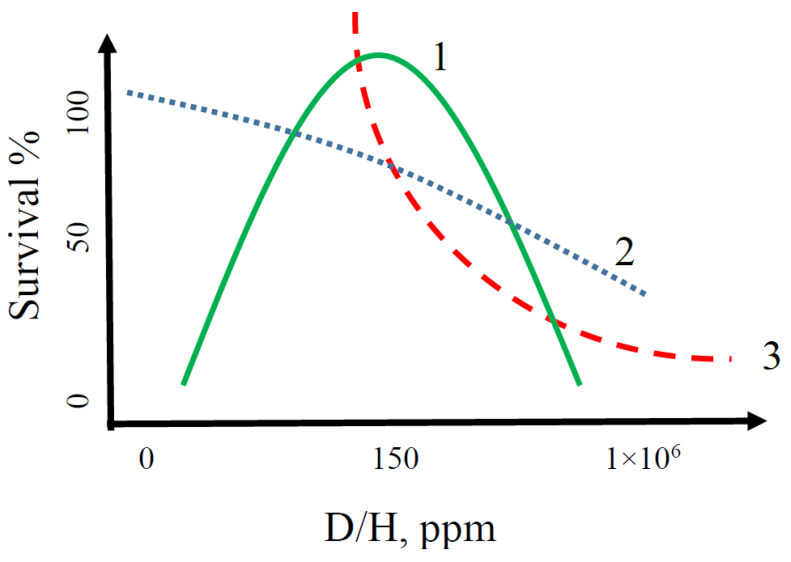
Possible kinetic model diagram of the vital activity of high-level biological taxons depending on the D/H ratio in the aquatic environment (1—animal; 2—bacteria; 3—plants).

Thus, there is a dependence on the D/H ratio and vital activity of living organisms! In our opinion, the diagram of the kinetic model of the vital activity of living organisms depending on the D/H ratio in the aquatic environment looks as follows (Figure 2).

The studies allowed to determine the sensitivity of some different taxons to changes in the content of deuterium in the aquatic environment. With some choice of taxons to determine the sensitivity to deuterium, we can give a preliminary assessment of the quality of the aquatic environment in relation to the D/H isotopic composition, in which the organism is located. It should be noted that several other authors independently obtained results similar to our data on the sensitivity of different taxons to deuterium concentrations for various vital indicators [27,38,45,46,47,48].

Since other authors previously presented data on changes in metabolism depending on the ratio of deuterium/protium, we did not focus on this. We tried to combine the data and describe the possible mechanism of metabolic alteration through the chirality of substances. Thus, the studies of bacteria were carried that showed a high sensitivity to the deuterated water [49,50]. Earlier studied the metabolism of the symbiotic organism medusomycetes (tea fungus) and the influence of D_2_O on its development [51]. Recent studies on the effects of low concentrations of deuterium on rats and mice show that the organism accelerates its metabolism [43,52,53,54]. In this regard showed that one could use ddw water to test inhibitors of *de novo* lipogenesis (DNL) by performing back-to-back studies in higher species, treat nonhuman primates with platensimycin, an inhibitor of fatty acid synthase [55]. Broad varieties of morphological and physiological changes were observed in deuterium-treated cells and organisms, including changes in fundamental processes such as cell division or energy metabolism [56]. However, it should be noted that we showed before how an excessive deficiency or an excess of deuterium can cause cytotoxic effects at the cell level [23,57]. Therefore, additional research of the use of water with a reduced deuterium content is required.

Taking into account many evidence of the kinetic isotope effect (KIE) for pharmaceutical substances in waters with different D/H ratios, it can be predicted that deuterium becomes a medical and chemical tool. Deuterated water is used as an inhibitor in medicines [58], which allows effective fighting against malignant tumors. This effect was also noted earlier in the deuterium-depleted water [39,40], although drugs based on such water have not yet been registered. The effect of deuterium on the kinetic effects of biotransformation of pharmaceutical substances may have significantly lower rates of metabolism, and hence a longer half-life. More and more deuterated compounds are characterized as promising substances for possible use as drugs. The first patents for deuterated substances were issued in the United States in the middle of the last century [59,60]. Such an example is deutetrabenazine (d_6_-tetrabenazine) approved by the Food and Drug Administration (FDA) in 2017 [61]. Two independent research groups reported a decrease in the biotransformation rate of d_2_-tyramine and d_3_-morphine compared to the parent proton-containing compounds [62,63].

Special attention should be paid to the mechanism of action of deuterium in living organisms. Several authors cite various theories [6,11,18,44,48,50], but they all boil down to the fact that the ratio of hydrogen isotopes modifies the metabolism in living organisms or their cells. Studied and considered different mechanisms of deuterated molecules action in living organisms: deuterium can make stronger chemical bonds than hydrogen and influence on the stretch frequency of bonds. Each C-H bond has a slightly different stretch frequency clustered around approximately 3.000 cm^−1^. Deuteration reduces the stretch frequencies by approximately the square root of 2.000 to 2.100 to 2.200 cm^−1^ [64]. Perhaps, in our opinion, the key mechanism is the chirality of substances in methylene groups with deuterium or protium in their structure. Recent data with a single ^2^H/^1^H replacement in DNA confirm this mechanism [65]. Therefore, further experimental data will help to solve the mechanism of action of deuterated or deuterium-depleted aqueous media on living organisms.

All of the above leads to the conclusion that the range of natural concentrations of deuterium in water is the most optimal for some living organisms on Earth, which apparently was formed over millions of years of evolution. Many organisms of different taxons are highly sensitive to high or low deuterium content in water. However, some taxons—for example, microorganisms—do not show statistically significant sensitivity to a change in the isotopic composition of water toward a decrease in deuterium compared with natural water. All this allowed us to assume that maybe the D/H ratio is a taxonomic determining factor for animals and plants, which presented in scheme Figure 2.

## 3. Materials and Methods

### 3.1. Physico-Chemical Analysis of Water with Different Deuterium Contentss

We used initial water samples with different deuterium contents in our work: deuterium-depleted water (DDW, ddw) with D/H = 5 ppm (Sigma-Aldrich, St. Louis, MO, USA); deuterated water D_2_O = 99 abs. at.% (Sigma-Aldrich). To study the effect of deuterium on the biological properties of organisms, media with different concentrations of deuterium were prepared by diluting deuterium-depleted and deuterated waters. As a control, water with the D/H ratio of 140 ppm was used. According to the physical indicators [8,17] and microelement composition, deuterium-depleted and deuterated waters had no difference, except for the deuterium content—this excluded the influence of multifactoriality in the system from the point of view of physico-chemical components for all compared groups. The method was described in more detail earlier [17]. The deuterium content was monitored using multipass laser absorption spectroscopy on Isotopic Water Analyzer-912-0032 (Los Gatos Research Inc., San Jose, CA, USA).

The chemical analysis of water with different deuterium contents was carried out by inductively coupled plasma mass spectrometry using an Agilent 7500CE ICP-QMS instrument (Agilent Technologies, Santa Clara, CA, USA); the method was described in more detail earlier [17,22]. To calibrate the instrument, calibration solutions were used in a wide range of element concentrations (from 0.1 μg/dm^3^ to 100 μg/dm^3^), they were prepared according to international reference sample (ISS) 2.74473.0100 ICP Multi Element Standard Solution XXI Certi PUR^®^. ISS contains the following elements: Ag, Al, As, Ba, Be, Bi, Ca, Cd, Co, Cr, Cs, Cu, Fe, Ga, In, K, Li, Mg, Mn, Na, Ni, Pb, Rb, Se, Sr, Tl, U, V, Zn, Hg. In deionized, deuterium-depleted and deuterated waters, the concentration of all elements did not exceed the upper detection limit (detection limit is 0.1–10 ppm).

The contents of Cd, Cu, Ni, Pb, Mn, Zn, Cr, Al, Fe, As, V were determined by atomic absorption spectrometry (AAS) method using an atomic absorption spectrometer Spectr AA-800 (Varian Inc., Palo Alto, CA, USA) with electrothermal atomization and the Zeeman effect according to the manufacturer’s protocol with modifications according to the results of the international intercalibration with the MEL laboratory of the IAEA (Monaco). The method was described in more detail earlier [17,22]. The source of radiation was single element hollow cathode lamps Spectr AA (Varian Inc., Palo Alto, CA, USA). The current of the lamps for elements Ni, Cu, Hg, Cd—4.0 mA; for Al, As—10 mA; Mn, Zn—5.0 mA; for Cr, V, Pb—7.0 mA, for Co, Sn—7 mA. The slit width of the monochromator was 0.5 nm when measuring Al, Ni, Cu, Zn, Hg; 0.2 nm for Cr, Fe, Mn, V, Co, Sn; 1.0 nm for Cd, Pb, As. The baseline correction mode and hot injection were carried out −80 °C. The following wavelengths (resonance lines) and modifiers were used: Al—256.8 nm, Mg(NO_3_)_2_; Ni—232.0 nm, Mg(NO_3_)_2_; Cr—429.0 nm, Mg(NO_3_)_2_; Mn—403.1 nm, Mg(NO_3_)_2_; Fe—386.0 nm, Mg(NO_3_)_2_; Cu—327.4 nm, Pd(NO_3_)_2_; Zn—307.6 nm, Mg(NO_3_)_2_, As—193.7 nm, Pd(NO_3_)_2_ + Mg(NO_3_)_2_; Sn—286.3 nm, Mg(NO_3_)_2_ + NH_4_H_2_PO_4_; V—318.5 nm; Co—242.5 nm, Pd(NO_3_)_2_; Cd—228.8 nm, Pd(NO_3_)_2_ + Mg(NO_3_)_2_ + NH_4_H_2_PO_4_; Pb—283.3 nm, Pd(NO_3_)_2_ + Mg(NO_3_)_2_ + NH_4_H_2_PO_4_. The relative standard deviation in the determination with a confidence level of 0.95 did not exceed 20%. The composition of the reference sample is given in [66].

### 3.2. Microbiological Methods

The method was previously described in more detail [1]. *Escherichia coli* 1257 strain was used in microbiological studies, which was obtained from the Research Institute for Standardization and Control of Medical Biological Preparations (Moscow, Russia). Bacteria from nutrient agar were inoculated into meat-peptone broth (MPB) and incubated in a temperature-controlled chamber at 37 °C for 18 h. The suspension was centrifuged at 7000 rpm for 15 min. The sedimental liquid was removed, and the sediment obtained was washed three times with sterile saline (0.9% NaCl) during the centrifugation. After that, the sediment was resuspended in the same physiological saline to a density of 1 × 10^8^ colony forming units (CFU) in 1 cm^3^. The corresponding aliquot of the initial suspension of *E. coli* was introduced into the test medium until obtaining the required microorganism density of 1 × 10^5^ CFU/cm^3^ [1].

The survival of microorganisms was determined by the presence of CFU when plating the selected samples on Endo agar medium. Petri dishes with medium were incubated in a temperature-controlled chamber at 37 °C for 18–24 h with subsequent counting of grown colonies. The result was expressed as the ratio of the logarithm of the test microorganism concentration, which remained in the solution (N*_t_*), to its initial count (N_0_).

### 3.3. Peptidomics of Moss Physcomitrella Patens

The method was described in more detail earlier [67].

#### 3.3.1. Sprouting of Moss Gametophores

*P. patens* moss gamethophores (*Gransden* strain) were grown on Knop modified agar medium (PPNO_3_ medium) in Petri dishes (diameter 9 cm) under white light from F96T12/GRO/VHO/WS fluorescent tubes with photon flux of 61 μM/(m^2^·s) under conditions of a 16-h photoperiod at 26 °C. Media based on water with various concentrations of deuterium were prepared for the study by diluting deuterium-depleted and deuterated waters. The gametophore groups were divided with a tweezer into fragments containing 4 to 5 shoots and transplanted onto fresh nutrient medium. 1 month old gametophores were used for the proteomic analysis.

#### 3.3.2. Isolation of Protein from Moss Tissues

The extraction of proteins from moss gametophores was carried out according to the method adapted for the *P. patens* moss [37]. Making of protein preparations from the gametophores: 1 month old shoots of moss were cut with a scalpel at a height of 1 mm from the surface of the agar medium and transferred with a tweezer to a porcelain mortar pre-cooled to −70 °C, in which they were immediately frozen with liquid nitrogen. Then, frozen shoots of moss were crushed to fine powder using a pre-cooled pestle (−70 °C). The plant material was poured with a 10% solution of 3-chloroacetic acid (TCA) in acetone with 0.07% dithiothreitol (DTT), cooled to −20 °C, then the material was incubated for 1 h at the same temperature to realize the protein precipitation. The protein precipitate suspension was centrifuged at 30,000× *g* for 15 min at 4 °C. The protein precipitate was poured with acetone cooled to −20 °C and containing 0.07% DTT, 1 mM PMSF, 2 mM EDTA, and shaken vigorously to remove pigments and lipids from the preparation. Then the protein precipitate suspension was centrifuged under the same conditions and the described procedure of washing the protein preparation with acetone was repeated two more times. Samples of protein preparations in centrifuge plastic tubes (2 mL) were dried in a vacuum centrifuge and stored at −70 °C for 10 days max (until the time of carrying out 2-dimensional gel electrophoresis).

#### 3.3.3. 2D Electrophoresis of Proteins

Two-dimensional separation of proteins was carried out according to the procedure [68] with some minor modifications. Upon completion of electrophoresis, the gels were labeled and stained with silver with thiosulfate [69]. The images of the silver stained gels were obtained using an Epson Perfection 4990 scanner and analyzed using the PDQuest 8.0 software (BioRad, Hercules, CA, USA).

#### 3.3.4. Trypsin Hydrolysis of Proteins

Proteins separated by 2D electrophoresis were hydrolyzed in PAGE pieces according to the technique proposed by Shevchenko and colleagues [70] with minor modifications.

#### 3.3.5. MALDI Mass Spectrometry

Tryptic peptides were extracted from the gel using 0.5% solution of 3-fluoroacetic acid (TFA) in water. The resulting extract in a volume of 0.20 μL was mixed with 0.25 μL of 2% solution of 2,5-dihydroxybenzoic acid in 30% solution of acetonitrile containing 0.5% glacial acetic acid by volume on a steel target, which was further dried in air at 23 °C for 30 min The peptides were analyzed on an Ultraflex-TOF/TOF time-of-flight mass spectrometer (Bruker Daltonics, Hamburg, Germany) equipped with a UV laser (337 nm). Positive ions were detected in the reflectron mode at voltages: at the ion source IS1—25 kV, IS2—21.75, 9.5 kV, reflectron Ref1—26.43 kV, Ref2—13.80 kV. Ions were detected at *m*/*z* = 700–4000. The peaks of trypsin autolytic fragments (*m*/*z* = 842.508, 1045.563, 2211.093), as well as keratin (*m*/*z* = 1475.780) and other impurities, which were excluded from the final lists of detected masses, were used as an internal reference.

#### 3.3.6. Analysis of Mass Spectrometry Results

Mass spectra were processed using the Flex Analysis 2.4 software (Bruker Daltonics). Smoothing by the Savitzky-Golay algorithm [71] (width 0.1 *m*/*z*, 1 cycle) and subtraction of the baseline according to the algorithm were applied to the mass spectra, Convex Hull [16]. The following peak detection parameters were used: peak detection algorithm—SNAP, signal-to-noise ratio—6, spectrum quality threshold—100. The search and identification of proteins by the “peptide imprint” method in the database of the nuclear genome of moss *P. patens* (fasta format, version 1.1, http://genome.jgi’psf.org/Phypa1_1/Phypa1_1.home.html) was performed using the MASCOT software package (local version 2.1.03, Matrix Science, Boston, MA, UK). In this case, the following search parameters were used: mass determination accuracy—100 ppm, possible post-translational modifications—methionine oxidation. Proteins with the probabilistic parameter MOWSE score higher than 58, which was considered the threshold for the base of the *P. patens* moss genome used, were considered reliably identified (95%, *p* > 0.05). An additional criterion for the reliability of protein identification was the coincidence of its molecular weight and/or isoelectric point with the corresponding parameters determined experimentally or predicted theoretically for the corresponding protein or its analogues.

#### 3.3.7. Liquid Chromatography-Mass Spectrometry (LC-MS/MS)

Peptide preparations obtained by trypsinolysis of proteins separated by 2D electrophoresis were analyzed on the Esquire 6000 plus mass spectrometer (Bruker Daltonics) equipped with a nanospray ion source with a quadrupole ion trap as a mass analyzer. The instrument is online mated with an Ultimate LC Packings nanochromatograph and Famos LC Packings sampling system (Dionex, Sunnyvale, CA, USA). The chromatographic part of the installation is two series-connected columns, between which there was an electromagnetic valve. Column No. 1 (100 μm × 3 cm) filled with the Poros R2 polymer phase (hydrophobic polymer phase with a large pore diameter, analog of C8) was used for preliminary concentration of the sample and its desalination. The second column (75 μm × 25 cm) filled with a Phenomenex sorbent (C18, grain size 5 μm, pore diameter 300 E) was used directly to separate a desalted mixture of tryptic peptides. The chromatographic separation on the system described above was carried out at a flow rate of 200 μL/min (up to the splitter). In this case, the actual flow rate during desalination was of 900 on average, and 200 μL/min during the separation. Peptides were separated using a linear gradient of “5–60%” solution of 75% acetonitrile, 25% isopropanol in 0.1% formic acid for 48 min. The measurements were carried out at *m*/*z* = 300–2500 with the “trap optimization mass” of 700. Tandem experiments were carried out only with ions, the charge number of which was 2, with an intensity above the threshold. The resulting lists of determined masses were sent to the MASCOT search engine. The search was carried out in the base of the nuclear genome of moss JGI (http://genome.jgi’psf.org/Phypa1_1/Phypa1_1.home.html), as well as in database NCBI (http://www.ncbi.nlm.nih.gov//genomes/geblast.cgi?bact=off&gi=5880) with the *P. patens* taxon sampling. The search results were verified using the Scaffold software package (version 01_07_00) (http://www.pro’teomesoftware.com) to confirm the correct determination of proteins and the identification of identical records when searching in two databases. Proteins identified by two or more peptides with a probability of at least 95% were left in the final list.

#### 3.3.8. 2D Electrophoresis of Proteins with Differential Staining (DiGE)

Total preparations of proteins isolated from moss gametophores were dissolved in a water buffer mixture (8 M urea (Amersham, St. Louis, MO, USA), 2 M thiourea (Amersham, St. Louis, MO, USA), 0.3% Chaps (Amersham), 0.1% Nonidet P_40 (Fluka, Munich, Germany), 10 mM Tris_HCl (Amersham), pH 8.0) for chromatography (Merck, Kenilworth, NJ, USA). Then, the samples were centrifuged at 14,000× *g* for 15 min at room temperature 25 °C. The protein concentration was measured by the Bradford protein assay using bovine serum albumin (BSA) as a reference (BioRad). Isoelectrofocusing and the second direction of electrophoresis were carried out according to the standard method. Scanning of the obtained gels was performed on a Typhoon instrument (Amersham) in the 600 PTM mode. After the fluorescence analysis, the gels were stained with silver according to the procedure described above.

### 3.4. Germination and Accumulation of Minerals in Callisia fragrans L.

The method was described in more detail earlier [72,73].

#### 3.4.1. Materials and Method at Germination of *C. fragrans* L.

To study the accumulation of minerals in waters with different D/H ratios, the leaves of the basket plant were used in the work (*Callisia fragrans*, fam. *Commelinaceae*). The plants were grown in climatic chambers (MIR-3, Ukraine, Russia) at 20 °C and relative humidity of 60%. The germination procedure was consistent with the methodology [73] with minor modifications. The following composition of the solution was used to saturate the shoots of the plant with a zinc complex: zinc glycinate (Zn (II) + Gly 1 mM + 20 mM), respectively (the experiment included three groups of plants: 1—germinated in water with natural isotopic composition; 2—in water with natural isotopic composition + Gly complex, 3—in deuterium-depleted water + Gly complex. Shoots were germinated in the prepared solution for 14 days. On the 12th day of the experiment and on the 14th day, the accumulation of elements in the lamina of the plant was observed. The lamina was divided into three parts of 2 cm: lower, medium and upper.

The fragrant callisia leaves were dried under mild (natural) conditions at the temperature of 25 °C for 5–7 days until a constant mass was achieved by weighing the sample once a day. Weighing was carried out on an ATL-80d4 analytical balance (Acculab, Vernon Hills, IL, USA). When fully dried, the leaves should be easy to grind into powder. Weight of dry raw material sample of leaves was 0.1576 g

#### 3.4.2. Dry Raw Material Analysis

The determination of chemical elements in the dried plant material was carried out without destroying the sample using a Shimadzu EDX-7000 X-ray fluorescence dispersion spectrophotometer (Shimadzu, Kyoto, Japan) based on a thermoelectrically cooled silicon drift detector equipped with the PCEDX-Navi software (Kyoto, Japan). The setup measurement conditions: current 100 μA, closed cuvette, 10 mm collimator, air medium, mylar and polypropylene films. The IAEA reference sample NIST SRM 2976 was used to calibrate the results. All results are presented for three repetitions with a confidence level of 95%. Each experimental repetition had three complete reproduction cycles of the entire experiment. The studies were carried out by the quantitative change of elements: Zn, Mn, Fe.

### 3.5. Determination of Biological Activity Using a Unicellular Biosensor Spirostomum ambiguum

The method was described in more detail earlier [19]. To determine the biological activity of a unicellular free-living eukaryotic organism of *S. ambiguum* ciliates in water with a modified D/H isotopic composition, the Spirotox method was used [19]. Observation of the biosensor was carried out using a binocular microscope MBR-10 (Altami, Saint Petersburg, Russia). To maintain a constant temperature in the medium, the Lauda A6 thermostat (Lauda-Königshofen, Germany) was used. As a result, of the study, the lifetime was determined and the activation energy (Ea) of the biosensor was found for different deuterium contents in water with a change in temperature conditions.

### 3.6. Determination of Embryotoxicity of Fish

A group of mature individuals *Brachydanio rerio* (*Hamilton, 1822*) was used in the studies. The conditions of keeping, feeding, and spawning of fish were described earlier [74]. Cultivation media were prepared according to the standard protocol based on deuterium-depleted, deuterated water and water. All reagents used were from the Sigma-Aldrich company, USA.

At the embryogenesis stages of blastomeres 4–32, the fertilized and non-fertilized eggs were separated from each other and transferred to the test aqueous solution. Only fertilized eggs were used for the experiments. The embryos were placed in Petri dishes of 7 cm diameter filled with the test solution. Each dilution was tested on at least 10 embryos at the rate of 2 mL of solution per one egg. The experiment was triplicated for each group. The control was in parallel. The results were considered reliable if no more than 10% of the embryos died in the control.

The embryos were observed for 72 h at the temperature of 26 °C in climatic chambers (MIR-3, Ukraine, Russia), the light regime corresponded to the alternation of day and night. At different stages of embryo development, the parameters indicating an embryogenesis developmental disorder were recorded [74].

The embryos were observed through an MBR-10 binocular microscope (Altami, Saint Petersburg, Russia) using a Canon PowerShot G9 digital camera with data and photo processing by MVideo and AxioVision software (White Plains, NY, USA). During the incubation, the parameters of the studied solutions were recorded: concentration of dissolved oxygen—using the Azha-101M oxygen meter (Altami), pH values—using a portable pH-meter pH-150M (Altami). In the studies, the indicators of the aquatic environment corresponded to the optimal parameters of the life of aquatic organisms—the oxygen level corresponded to 5–8 mg O_2_/cm^3^, and the pH value was within the range of 6.5–8.5.

### 3.7. Ethical Standards of Studies in Animals

All the studies were carried out in accordance with international ethics requirements approved by Directive 2010/63/EU for experiments using animals.

### 3.8. Statistical Data Processing

All statistical data processing was performed using Student’s *t*-test, as well as using the one-way analysis of variance (ANOVA) in the Origin Pro software. The differences were considered statistically significant at *p* < 0.05.

## 4. Conclusion

We determined the reactions of some major taxonomic groups of organisms to changes in the ratio of D/H isotopes in the aquatic environment. It was found that the studied organisms react differently to both high and low concentrations of deuterium in aqueous solutions, reducing or increasing their biological activity, perhaps by variety metabolism (moss peptidom changes). Perhaps ratio of D/H this is a determining taxonomic factor for living organisms. This theory is supported by the developed kinetic model and the presented diagram of biocatalytic reactions of the vital activity of living organisms depending on the D/H ratio in aqueous solutions.

## Figures and Tables

**Table 1:** Some physico-chemical properties of water with different heavy isotope contents H12(D) (T = 20.00 ± 0.04 °C) [8]. (*—according to the LALLS method data).

No.	Physico-Chemical Parameter	Water D/H = 4 ± 0.9 ppm (ddw)	High-Resistance Water of Natural Isotopic Composition D/H = 140 ± 0.9 ppm	D_2_O 99.9%
1	Surface Tension σ, mN/m	75.172	72.860	67.800
2	Kinematic Viscosity, mm^2^/s	0.987	1.012	1.274
3	Density *, g/cm^3^ * O^18^/O^16^ = 757 ppm; t° = 25 ± 0.05 °C	0.9969	0.9982	1.1042
4	Freezing Point, T °C	−1.5	0	+3.8
5	D_l_—self-diffusion coefficient, 10^9^, m^2^s^−1^	0.63	0.46	0.52
6	Spin-spin Relaxation Time of the Water Proton t, s	0.347	2.000	−
7	Volume concentration * of density inhomogeneities, vc (%)	0.20	1.00	0.18
8	Obscuration * due to laser light scattering, laserobscuration (λ = 633 nm)	0.003	0.020	0.005

**Table 2:** The influence of the changed D/H isotopic composition in water on the values of vital indicators of different taxonomic groups of organisms. (Control—indicators in water with the natural ratio of deuterium/protium (140 ppm); *—death).

D/H, ppm	Organisms, Indicators				
	Bacteria	Plants		Animals	
		*Bryopsida*	*Magnoliophyta*	*Protozoa*	*Metazoa (Fish)*
	*E.coli*, CFU	*P. patens*, growth	*C. fragrans* L., accumulation of Zn	*S. ambiguum*, survival	*B. rerio*, embryo toxicity (survival)
10	100%	70%	4 × 10^3^%	5%	80%
140	100%	100%	100%	100%	100%
D_2_O	35%	0% *	0% *	1%	50%

**Table 3:** Zinc accumulation factor (k) in *C. fragrans* leaves after incubation with zinc glycinate solutions in ordinary MilliQ water (D/H = 142 ppm) and (ddw) (D/H = 12 ppm). k = [Zn] after incubation with Zn (II) glycinate/[Zn] before incubation.(where [Zn] is total concentration of the element). Measurements were carried out for the lower (in the petiole) part of the leaf blade. Data for a seven-day incubation are presented. *—the value corresponds to the absolute content of the element Zn—1.5 mg per 1 g for dry weight.

Element	k	
	**Zn-Gly**	**Zn-Gly in ddw**
Zn	40 ± 10	160 ± 10 *
Mn	1 ± 0.3	0.7 ± 0.2
Fe	3 ± 1	10 ± 2 *

**Table 4:** Changes in the moss peptidom during the sprouting on DDW (peptide names are given according to international nomenclature [36]). “+”—present; “−”—absent.

Item No.	Peptide Code and Characterization of the Proteins	Sprouting on MilliQ	Sprouting on DDW
1	A9RAW3_PHYPA Uncharacterized protein OS = Physcomitrella patens subsp. patens GN = PHYPADRAFT_158431 PE = 3; SV = 1	+	−
2	A9REY5_PHYPA Predicted protein OS = Physcomitrella patens subsp. patens GN = PHYPADRAFT_113064 PE = 3; SV = 1	+	−
3	A9RFC9_PHYPA Predicted protein OS = Physcomitrella patens subsp. patens GN = PHYPADRAFT_113309 PE = 3; SV = 1; D2XNF3	+	−
4	A9RJM3_PHYPA NADH-cytochrome b5 reductase OS = Physcomitrella patens subsp. patens GN = PHYPADRAFT_115361 PE = 3; SV = 1	+	−
5	A9RKT5_PHYPA Predicted protein OS = Physcomitrella patens subsp. patens GN = PHYPADRAFT_175733 PE = 4; SV = 1; A9S7I6	+	−
6	A9RLI4_PHYPA Predicted protein OS=Physcomitrella patens subsp. patens GN = PHYPADRAFT_203665 PE = 4; SV = 1	+	−
7	A9RQ53_PHYPA Predicted protein OS = Physcomitrella patens subsp. patens GN = PHYPADRAFT_62373 PE = 4; SV = 1	+	−
8	A9SNF4_PHYPA Serine/threonine-protein phosphatase OS = Physcomitrella patens subsp. patens GN = PHYPADRAFT_186751 PE = 3; SV = 1; A9S099	+	−
9	A9RR01_PHYPA Predicted protein OS = Physcomitrella patens subsp. patens GN = PHYPADRAFT_160535 PE = 4; SV = 1	+	−
10	A9RWR3_PHYPA Predicted protein OS = Physcomitrella patens subsp. patens GN = PHYPADRAFT_206327 PE = 4; SV = 1	+	−
11	A9RXH5_PHYPA Predicted protein (Fragment) OS = Physcomitrella patens subsp. patens GN = PHYPADRAFT_20993 PE = 4; SV = 1; A9RXH0	+	−
12	A9RXS6_PHYPA ATPase ASNA1 homolog OS = Physcomitrella patens subsp. patens GN = PHYPADRAFT_206681 PE = 3; SV = 1	+	−
13	A9RY14_PHYPA Predicted protein OS = Physcomitrella patens subsp. patens GN = PHYPADRAFT_161687 PE = 3; SV = 1	+	−
14	A9RZM5_PHYPA Malic enzyme OS = Physcomitrella patens subsp. patens GN = PHYPADRAFT_179808 PE = 3; SV = 1	+	−
15	A9S3P3_PHYPA Predicted protein OS = Physcomitrella patens subsp. patens GN = PHYPADRAFT_123533 PE = 4; SV = 1; Q05KM6	+	−
16	A9S3W0_PHYPA Predicted protein (Fragment) OS = Physcomitrella patens subsp. patens GN = PHYPADRAFT_5967 PE = 3; SV = 1	+	−
17	A9SVS7_PHYPA Predicted protein (Fragment) OS = Physcomitrella patens subsp. patens GN = PHYPADRAFT_19427 PE = 4; SV = 1; A9S6D6	+	−
18	A9S6F0_PHYPA Predicted protein OS = Physcomitrella patens subsp. patens GN = PHYPADRAFT_163089 PE = 4; SV = 1	+	−
19	A9SDC7_PHYPA Predicted protein OS = Physcomitrella patens subsp. patens GN = PHYPADRAFT_183678 PE = 4; SV = 1	+	−
20	A9SE00_PHYPA Predicted protein OS = Physcomitrella patens subsp. patens GN = PHYPADRAFT_164448 PE = 4; SV = 1	+	−
21	A9SE49_PHYPA Predicted protein OS = Physcomitrella patens subsp. patens GN = PHYPADRAFT_211358 PE = 4; SV = 1	+	−
22	A9SJI0_PHYPA Predicted protein OS = Physcomitrella patens subsp. patens GN = PHYPADRAFT_213039 PE = 3; SV = 1	+	−
23	A9SNN0_PHYPA Predicted protein OS = Physcomitrella patens subsp. patens GN = PHYPADRAFT_186902 PE = 4; SV = 1	+	−
24	A9SPQ9_PHYPA Acyl carrier protein OS = Physcomitrella patens subsp. patens GN = PHYPADRAFT_110052 PE = 3; SV = 1	+	−
25	A9SRJ7_PHYPA Predicted protein OS = Physcomitrella patens subsp. patens GN = PHYPADRAFT_134103 PE = 4; SV = 1	+	−
26	A9SUV8_PHYPA Beta-amylase OS = Physcomitrella patens subsp. patens GN = PHYPADRAFT_107034 PE = 3; SV = 1	+	−
27	A9SZ47_PHYPA Predicted protein OS = Physcomitrella patens subsp. patens GN = PHYPADRAFT_217333 PE = 3; SV = 1	+	−
28	A9SZE9_PHYPA Xyloglucan endotransglucosylase/hydrolase OS = Physcomitrella patens subsp. patens GN = PHYPADRAFT_137834 PE = 3; SV = 1	+	−
29	A9SZY0_PHYPA Predicted protein OS = Physcomitrella patens subsp. patens GN = PHYPADRAFT_167354 PE = 4; SV = 1	+	−
30	A9T1V0_PHYPA Predicted protein (Fragment) OS = Physcomitrella patens subsp. patens GN = PHYPADRAFT_138980 PE = 4; SV = 1	+	−
31	A9T469_PHYPA 40S ribosomal protein S8 OS = Physcomitrella patens subsp. patens GN = PHYPADRAFT_191463 PE = 3; SV = 1	+	−
32	A9T7K4_PHYPA Predicted protein OS = Physcomitrella patens subsp. patens GN = PHYPADRAFT_110253 PE = 3; SV = 1	+	−
33	A9TE26_PHYPA Cysteine proteinase inhibitor OS = Physcomitrella patens subsp. patens GN = PHYPADRAFT_144213 PE = 3; SV = 1	+	−
34	A9TF90_PHYPA Predicted protein (Fragment) OS = Physcomitrella patens subsp. patens GN = PHYPADRAFT_144707 PE = 4; SV = 1	+	−
35	A9THA6_PHYPA Predicted protein OS = Physcomitrella patens subsp. patens GN = PHYPADRAFT_222325 PE = 4; SV = 1	+	−
36	A9THL7_PHYPA Predicted protein OS = Physcomitrella patens subsp. patens GN = PHYPADRAFT_170119 PE = 4; SV = 1	+	−
37	A9THY4_PHYPA Predicted protein (Fragment) OS = Physcomitrella patens subsp. patens GN = PHYPADRAFT_6064 PE = 4; SV = 1	+	−
38	A9TJ89_PHYPA Predicted protein OS = Physcomitrella patens subsp. patens GN = PHYPADRAFT_146390 PE = 3; SV = 1	+	−
39	A9TLJ0_PHYPA Carboxypeptidase OS = Physcomitrella patens subsp. patens GN = PHYPADRAFT_147350 PE = 3; SV = 1	+	−
40	A9TMY7_PHYPA Predicted protein OS = Physcomitrella patens subsp. patens GN = PHYPADRAFT_223748 PE = 3; SV = 1	+	−
41	A9TPH1_PHYPA Predicted protein OS = Physcomitrella patens subsp. patens GN = PHYPADRAFT_224194 PE = 4; SV = 1; A9U2L4	+	−
42	A9TQB7_PHYPA Predicted protein OS = Physcomitrella patens subsp. patens GN = PHYPADRAFT_148985 PE = 3; SV = 1	+	−
43	A9TUQ5_PHYPA Predicted protein OS = Physcomitrella patens subsp. patens GN = PHYPADRAFT_108826 PE = 4; SV = 1; A9T4Q4	+	−
44	Q8GU37_PHYPA Putative phosphatidylcholine-sterol acetyltransferase (Fragment) OS = Physcomitrella patens subsp. patens PE = 2; SV = 1; A9TVL8	+	−
45	A9TY79_PHYPA Predicted protein OS = Physcomitrella patens subsp. patens GN = PHYPADRAFT_199405 PE = 3; SV = 1	+	−
46	A9U536_PHYPA Predicted protein (Fragment) OS = Physcomitrella patens subsp. patens GN = PHYPADRAFT_23950 PE = 4; SV = 1; A9U5D9	+	−
47	Q1XGA6_PHYPA Cytochrome b OS = Physcomitrella patens subsp. patens GN = cob PE = 3; SV = 1	+	−
48	RR19_PHYPA 30S ribosomal protein S19, chloroplastic OS = Physcomitrella patens subsp. patens GN = rps19; PE = 3; SV = 1	−	+
49	A9SDA4_PHYPA Predicted protein OS = Physcomitrella patens subsp. patens GN = PHYPADRAFT_211044 PE = 3; SV = 1; A9TQF6	−	+
50	A9SE53_PHYPA Predicted protein OS = Physcomitrella patens subsp. patens GN = PHYPADRAFT_78151 PE = 4 SV = 1; A9SUI0	−	+
51	A9RXH3_PHYPA Predicted protein OS = Physcomitrella patens subsp. patens GN = PHYPADRAFT_121056 PE = 4; SV = 1	−	+
52	A9RQE4_PHYPA 40S ribosomal protein S30 (Fragment) OS = Physcomitrella patens subsp. patens GN = PHYPADRAFT_118030 PE = 3; SV = 1; A9RQD4	−	+
53	A9SBL7_PHYPA Predicted protein OS = Physcomitrella patens subsp. patens GN = PHYPADRAFT_183147 PE = 4; SV = 1	−	+
54	A9SF90_PHYPA Predicted protein (Fragment) OS = Physcomitrella patens subsp. patens GN = PHYPADRAFT_128900 PE = 4; SV = 1	−	+
55	A9T230_PHYPA Predicted protein OS = Physcomitrella patens subsp. patens GN = PHYPADRAFT_233069 PE = 1; SV = 1	−	+
56	A9SWB0_PHYPA Predicted protein OS = Physcomitrella patens subsp. patens GN = PHYPADRAFT_136325 PE = 4; SV = 1	−	+
57	A9SUQ5_PHYPA Predicted protein OS = Physcomitrella patens subsp. patens GN = PHYPADRAFT_84227 PE = 4; SV = 1	−	+
58	A9T6P0_PHYPA Predicted protein OS = Physcomitrella patens subsp. patens GN = PHYPADRAFT_168383 PE = 4; SV = 1	−	+
59	Q1L642_PHYPA Plasma membrane aquaporin OS = Physcomitrella patens subsp. patens GN = PIP1;1 PE = 3; SV = 1; A9RBK8	−	+
60	A9SKG1_PHYPA Predicted protein OS = Physcomitrella patens subsp. patens GN = PHYPADRAFT_131352 PE = 4; SV = 1	−	+
61	A9RGM6_PHYPA Predicted protein OS = Physcomitrella patens subsp. patens GN = PHYPADRAFT_114028 PE = 3; SV = 1	−	+
62	RK14_PHYPA 50S ribosomal protein L14, chloroplastic OS = Physcomitrella patens subsp. patens GN = rpl14; PE = 3; SV = 1	−	+
63	A9SG27_PHYPA Predicted protein OS = Physcomitrella patens subsp. patens GN = PHYPADRAFT_129247 PE = 3; SV = 1; A9RZ43	−	+
64	A9TEN6_PHYPA Predicted protein OS = Physcomitrella patens subsp. patens GN = PHYPADRAFT_169641 PE = 4; SV = 1	−	+
65	A9SZZ7_PHYPA Predicted protein OS = Physcomitrella patens subsp. patens GN = PHYPADRAFT_217573 PE = 4; SV = 1	−	+
66	A9RS97_PHYPA Predicted protein (Fragment) OS = Physcomitrella patens subsp. patens GN = PHYPADRAFT_118507 PE = 4; SV = 1; A9SZB8	−	+
67	A9SKW2_PHYPA Peptidylprolyl isomerase (Fragment) OS = Physcomitrella patens subsp. patens GN = PHYPADRAFT_49268 PE = 4; SV = 1; A9TGA1	−	+
68	A9U1T5_PHYPA Predicted protein (Fragment) OS = Physcomitrella patens subsp. patens GN = PHYPADRAFT_5105 PE = 4; SV = 1	−	+
69	A9U1Y5_PHYPA Predicted protein OS = Physcomitrella patens subsp. patens GN = PHYPADRAFT_173229 PE = 4; SV = 1	−	+
70	A9U341_PHYPA Predicted protein (Fragment) OS = Physcomitrella patens subsp. patens GN = PHYPADRAFT_8489 PE = 4; SV = 1	−	+
71	A9T9M8_PHYPA Predicted protein OS = Physcomitrella patens subsp. patens GN = PHYPADRAFT_142289 PE = 3; SV = 1	−	+
72	A9U096_PHYPA Predicted protein OS = Physcomitrella patens subsp. patens GN = PHYPADRAFT_153718 PE = 4; SV = 1; A9SU96	−	+
73	A9T027_PHYPA Predicted protein OS = Physcomitrella patens subsp. patens GN = PHYPADRAFT_86316 PE = 3; SV = 1	−	+
74	A9TA00_PHYPA Predicted protein OS = Physcomitrella patens subsp. patens GN = PHYPADRAFT_220250 PE = 3; SV = 1	−	+
75	Q5KSB5_PHYPA Germin-like protein OS = Physcomitrella patens subsp. patens GN = PpGLP6 PE = 2; SV = 1	−	+
76	A9S3L2_PHYPA Predicted protein OS = Physcomitrella patens subsp. patens GN = PHYPADRAFT_180874 PE = 3; SV = 1	−	+
77	A9RGB1_PHYPA Uncharacterized protein OS = Physcomitrella patens subsp. patens GN = PHYPADRAFT_104506 PE = 3; SV = 1	−	+
78	A9S1H8_PHYPA Predicted protein (Fragment) OS = Physcomitrella patens subsp. patens GN = PHYPADRAFT_44384 PE = 4; SV = 1; A9SQA7	−	+
79	A9TNC3_PHYPA Predicted protein OS = Physcomitrella patens subsp. patens GN = PHYPADRAFT_196700 PE = 4; SV = 1	−	+
80	A9RY38_PHYPA Purple acid phosphatase OS = Physcomitrella patens subsp. patens GN = PHYPADRAFT_121352 PE = 3; SV = 1	−	+
81	A9TJF9_PHYPA Predicted protein (Fragment) OS = Physcomitrella patens subsp. patens GN = PHYPADRAFT_33800 PE = 4; SV = 1	−	+
82	A9SKS5_PHYPA Predicted protein OS = Physcomitrella patens subsp. patens GN = PHYPADRAFT_186065 PE = 4; SV = 1; A9RPK4	−	+
83	A9SIT1_PHYPA Predicted protein OS = Physcomitrella patens subsp. patens GN = PHYPADRAFT_165118 PE = 4; SV = 1	−	+
84	A9U068_PHYPA Predicted protein OS = Physcomitrella patens subsp. patens GN = PHYPADRAFT_227113 PE = 4; SV = 1	−	+
85	A9T8V9_PHYPA Predicted protein OS = Physcomitrella patens subsp. patens GN = PHYPADRAFT_89614 PE = 4; SV = 1; B7SB99	−	+
86	A9TJZ6_PHYPA Predicted protein OS = Physcomitrella patens subsp. patens GN = PHYPADRAFT_146721 PE = 4; SV = 1	−	+

## References

[1] Goncharuk V.V., Pleteneva T.V., Grebennikova T.V., Syroeshkin A.V., Uspenskaya E.V., Antipova N.V., Kovalenko V.F., Saprykina M.N., Skil’Skaya M.D., Zlatskiy I.A. (2018). Determination of Biological Activity of Water Having a Different Isotope Ratio of Protium and Deuterium. J. Water Chem. Technol..

[2] Lobyshev V.N., Kalinichenko L.P. (1978). Isotopic Effects in Biological Systems.

[3] McCartney D., Desbrow B., Irwin C. (2017). Post-exercise Ingestion of Carbohydrate, Protein and Water: A Systematic Review and Meta-analysis for Effects on Subsequent Athletic Performance. Sports Med..

[4] Said H.M. (2011). Intestinal absorption of water-soluble vitamins in health and disease. Biochem. J..

[5] Goncharuk V.V. (2010). Science about Water.

[6] Basov A., Fedulova L., Baryshev M., Dzhimak S. (2019). Deuterium-Depleted Water Influence on the Isotope 2H/1H Regulation in Body and Individual Adaptation. Nutrients.

[7] Bowen G.J., Winter D.A., Spero H.J., Zierenberg R., Reeder M.D., Cerling T.E., Ehleringer J.R. (2005). Stable hydrogen and oxygen isotope ratios of bottled waters of the world. Rapid Commun. Mass Spectrom..

[8] Goncharuk V.V., Lapshin V.B., Burdeynaya T.N., Pleteneva T.V., Chernopyatko A.S., Atamanenko I.D. (2011). Physico-chemical properties and biological activity of water, lean on heavy isotope. Chem. Technol. Water.

[9] Lewis G.N. (1934). Biology of heavy water. Nature.

[10] Timmins G.S. (2014). Deuterated drugs: Where are we now?. Expert Opin. Ther. Patents.

[11] Boros L.G., D’Agostino D.P., Katz H.E., Roth J.P., Meuillet E.J., Somlyai G. (2016). Submolecular regulation of cell transformation by deuterium depleting water exchange reactions in the tricarboxylic acid substrate cycle. Med. Hypotheses.

[12] Robins R.J., Remaud G.S., Billault I. (2012). Natural mechanisms by which deuterium depletion occurs in specific positions in metabolites. Eur. Chem. Bull..

[13] Cleland W.W. (2003). The use of isotope effects to determine enzyme mechanisms. J. Biol. Chem..

[14] Strekalova T., Evans M., Chernopiatko A., Couch Y., Costa-Nunes J.P., Cespuglio R., Chesson L., Vignisse J., Steinbusch H.W., Anthony D.C. (2015). Deuterium content of water increases depression susceptibility: The potential role of a serotonin-related mechanism. Behav. Brain Res..

[15] Cărpinişan L., Petcu M.D., Petrovici S., Chiş C., Ghişe A., Zehan R. (2010). The Influence of deuterium depleted water on the hematocrit and the leukocyte formula in rats intoxicated with chromium. Sci. Pap. Anim. Sci. Biotechnol..

[16] Huynh M.H.V., Meyer T.J. (2004). Colossal kinetic isotope effects in proton-coupled electron transfer. Proc. Natl. Acad. Sci. USA.

[17] Syroeshkin A., Pleteneva T., Uspenskaya E., Zlatskiy I.A., Antipova N., Grebennikova T., Levitskaya O.V. (2019). D/H control of chemical kinetics in water solutions under low deuterium concentrations. Chem. Eng. J..

[18] Basov A., Fedulova L., Vasilevskaya E.R., Dzhimak S. (2019). Possible Mechanisms of Biological Effects Observed in Living Systems during 2H/1H Isotope Fractionation and Deuterium Interactions with Other Biogenic Isotopes. Molecules.

[19] Goncharuk V.V., Syroeshkin A.V., Zlatskiy I.A., Uspenskaya E.V., Orekhova A.V., Levitskaya O.V., Dobrovolskiy V.I., Pleteneva T. (2017). Quasi-chemical description of the kinetics of cell death Spirostomum ambiguum biosensor for biological activity of aqueous solutions. J. Water Chem. Technol..

[20] Atzrodt J., Derdau V., Kerr W.J., Reid M. (2018). Deuterium- and Tritium-Labelled Compounds: Applications in the Life Sciences. Angew. Chem. Int. Ed..

[21] Farthing D.E., Buxbaum N.P., Lucas P.J., Maglakelidze N., Oliver B., Wang J., Hu K., Castro E., Bare C.V., Gress R.E. (2017). Comparing DNA enrichment of proliferating cells following administration of different stable isotopes of heavy water. Sci. Rep..

[22] Syroeshkin A., Antipova N., Zlatska A., Zlatskiy I.A., Skylska M., Grebennikova T., Goncharuk V. (2018). The effect of the deuterium depleted water on the biological activity of the eukaryotic cells. J. Trace Elem. Med. Boil..

[23] Zlatska A.V., Vasyliev R.G., Gordiienko I.M., Rodnichenko A.E., Morozova M.A., Vulf M.A., Zubov D.O., Novikova S.N., Litvinova L.S., Grebennikova T.V. (2020). Effect of the deuterium on efficiency and type of adipogenic differentiation of human adipose-derived stem cells in vitro. Sci. Rep..

[24] Makarova M., Syroeshkin A., Maksimova T., Pleteneva T., Zlatskiy I., Antipova N., Skripnikov A., Dzhavakhyan M. (2019). D/H modification of plant peptides and microelements metabolome. FEBS Open Bio.

[25] Tsisanova E.S., Uspenskaya E.V., Pleteneva T.V., Syroeshkin A.V. (2010). Study of biological activity and D/H ratio of water with the aid of cellular biosensor spirostomum ambiguum. Trace Elem. Med..

[26] Chuparina E.V., Gunicheva T.N. (2004). State and issues of X-ray fluorescence analysis of herbal raw materials. Anal. Control.

[27] McCluney K.E., Sabo J.L. (2010). Tracing Water Sources of Terrestrial Animal Populations with Stable Isotopes: Laboratory Tests with Crickets and Spiders. PLoS ONE.

[28] Goncharuk V.V., Taranov V.V., Kurlyantseva A.Y., Syroeshkin A.V. (2015). Phase transition in waters with different content of deuterium. J. Water Chem. Technol..

[29] Rodin S., Rebellato P., Lundin A., Zubarev R.A. (2018). Isotopic resonance at 370 ppm deuterium negatively affects kinetics of luciferin oxidation by luciferase. Sci. Rep..

[30] Dzhimak S., Basov A.A., Baryshev M.G. (2015). Content of deuterium in biological fluids and organs: Influence of deuterium depleted water on D/H gradient and the process of adaptation. Dokl. Biochem. Biophys..

[31] Luo A.L., Zheng Y.L., Cong F.S. (2018). Research progress of biological effects of deuterium-depleted water. J. Shanghai Jiaotong Univ. (Med. Sci.).

[32] Trouiller B., Schaefer D.G., Charlot F., Nogué F. (2006). MSH2 is essential for the preservation of genome integrity and prevents homeologous recombination in the moss Physcomitrella patens. Nucleic Acids Res..

[33] Schaefer D.G. (2001). Gene targeting in Physcomitrella patens. Curr. Opin. Plant Biol..

[34] Mittmann F., Brücker G., Zeidler M., Repp A., Abts T., Hartmann E., Hughes J. (2004). Targeted knockout in Physcomitrella reveals direct actions of phytochrome in the cytoplasm. Proc. Natl. Acad. Sci. USA.

[35] Rensing S.A., Lang D., Zimmer A., Terry A., Salamov A., Shapiro H., Nishiyama T., Perroud P.-F., Lindquist E., Kamisugi Y. (2007). The Physcomitrella Genome Reveals Evolutionary Insights into the Conquest of Land by Plants. Science.

[36] Sarnighausen E., Wurtz V., Heintz D., Van Dorsselaer A., Reski R. (2004). Mapping of the Physcomitrella patens proteome. Phytochemisty.

[37] Cho S.H., Hoang Q.T., Kim Y.Y., Shin H.Y., Ok S.H., Bae J.M., Shin J.S. (2006). Proteome analysis of gametophores identified a metallothionein involved in various abiotic stress responses in Physcomitrella patens. Plant Cell Rep..

[38] Avila D.S., Somlyai G., Somlyai I., Aschner M. (2012). Anti-aging effects of deuterium depletion on Mn-induced toxicity in a *C. elegans* model. Toxicol. Lett..

[39] Somlyai G. (2002). Defeating Cancer. The Biological Effects of Deuterium Depletion.

[40] Somlyai G., Gyöngyi Z., Somlyai I., Boros L.G. (2016). Pre-clinical and clinical data confirm the anticancer effect of Deuterium depletion. Eur. J. Integr. Med..

[41] Zhang K., Toki H., Fujita Y., Ma M., Chang L., Qu Y., Harada S., Nemoto T., Mizuno-Yasuhira A., Yamaguchi J.-I. (2018). Lack of deuterium isotope effects in the antidepressant effects of (R)-ketamine in a chronic social defeat stress model. Psychopharmacology.

[42] Yavari K., Kooshesh L. (2019). Deuterium Depleted Water Inhibits the Proliferation of Human MCF7 Breast Cancer Cell Lines by Inducing Cell Cycle Arrest. Nutr. Cancer.

[43] Halenova T., Zlatskiy I.A., Syroeshkin A.V., Maximova T., Pleteneva T. (2019). Deuterium-Depleted Water as Adjuvant Therapeutic Agent for Treatment of Diet-Induced Obesity in Rats. Molecules.

[44] Goncharuk V.V., Syroeshkin A.V., Pleteneva T.V., Uspenskaya E.V., Levitskaya O.V., Tverdislov V.A. (2017). On the possibility of chiral structure-density submillimeter inhomogeneities existing in water. J. Water Chem. Technol..

[45] Basov A.A., Elkina A.A., Samkov A.A., Volchenko N.N., Moiseev A.V., Fedulova L.V., Baryshev M.G., Dzhimak S.S. (2019). Influence of Deuterium-Depleted Water on the Isotope D/H Composition of Liver Tissue and Morphological Development of Rats at Different Periods of Ontogenesis. Iran. Biomed. J..

[46] Rasooli A., Fatemi F., Hajihosseini R., Vaziri A., Akbarzadeh K., Malayeri M.R.M., Dini S., Foroutanrad M. (2019). Synergistic effects of deuterium depleted water and Mentha longifolia L. essential oils on sepsis-induced liver injuries through regulation of cyclooxygenase-2. Pharm. Boil..

[47] Vasilevskaya E.R., Akhremko A. (2019). Proteomic study of pig’s spleen. Potravin. Slovak J. Food Sci..

[48] Pedersen L.G., Bartolotti L., Li L. (2006). Deuterium and its role in the machinery of evolution. J. Theor. Boil..

[49] Xie X., Zubarev R.A. (2017). On the Effect of Planetary Stable Isotope Compositions on Growth and Survival of Terrestrial Organisms. PLoS ONE.

[50] Zubarev R.A. (2011). Role of stable isotopes in life—Testing isotopic resonance hypothesis. Genom. Proteom. Bioinform..

[51] Kutyshenko V.P., Iurkevich D.I. (2003). Effect of heavy water on metabolism of a symbiotic organism. Biofizika.

[52] Gyöngyi Z., Budán F., Szabó I., Ember I., Kiss I., Krempels K., Somlyai I., Somlyai G. (2013). Deuterium Depleted Water Effects on Survival of Lung Cancer Patients and Expression of Kras, Bcl2, and Myc Genes in Mouse Lung. Nutr. Cancer.

[53] Kravtsov A.A., Kozin S.V., Vasilevskaya E.R., Elkina A.A., Fedulova L.V., Popov K., Malyshko V.V., Moiseev A., Shashkov D.I., Baryshev M.G. (2018). Effect of Drinking Ration with Reduced Deuterium Content on Brain Tissue Prooxidant-Antioxidant Balance in Rats with Acute Hypoxia Model. J. Pharm. Nutr. Sci..

[54] Basov A.A., Kozin S.V., Bikov I.M., Popov K.A., Moiseev A.V., Elkina A.A., Dzhimak S.S. (2019). Changes in Prooxidant-Antioxidant System Indices in the Blood and Brain of Rats with Modelled Acute Hypoxia which Consumed a Deuterium-Depleted Drinking Diet. Biol. Bull..

[55] Previs S.F., Herath K., Nawrocki A.R., Rodriguez C.G., Slipetz D., Singh S.B., Kang L., Bhat G., Roddy T.P., Conarello S. (2018). Using [2H]water to quantify the contribution of de novo palmitate synthesis in plasma: Enabling back-to-back studies. Am. J. Physiol. Metab..

[56] Kselíková V., Vítová M., Bišová K. (2019). Deuterium and its impact on living organisms. Folia Microbiol..

[57] Zlatska A., Gordiienko I., Vasyliev R., Zubov D.O., Gubar O., Rodnichenko A.E., Syroeshkin A., Zlatskiy I.A. (2018). In Vitro Study of Deuterium Effect on Biological Properties of Human Cultured Adipose-Derived Stem Cells. Sci. World J..

[58] Pirali T., Serafini M., Cargnin S., Genazzani A.A. (2019). Applications of Deuterium in Medicinal Chemistry. J. Med. Chem..

[59] Tung R.D. (2016). Deuterium medicinal chemistry comes of age. Future Med. Chem..

[60] Timmins G.S. (2017). Deuterated drugs; updates and obviousness analysis. Expert Opin. Ther. Patents.

[61] Russak E., Bednarczyk E.M. (2018). Impact of Deuterium Substitution on the Pharmacokinetics of Pharmaceuticals. Ann. Pharmacother..

[62] Elison C., Rapoport H., Laursen R., Elliott H.W., De Vries H., Dreimanis A. (1961). Effect of Deuteration of N—CH3 Group on Potency and Enzymatic N-Demethylation of Morphine. Science.

[63] Sipes I.G., Gandolfi A.J., Pohl L.R., Krishna G., Brown B.R. (1980). Comparison of the biotransformation and hepatotoxicity of halothane and deuterated halothane. J. Pharmacol. Exp. Ther..

[64] Franco M.I., Turin L., Mershin A., Skoulakis E.M. (2011). Molecular vibration-sensing component in Drosophila melanogaster olfaction. Proc. Natl. Acad. Sci. USA.

[65] Basov A., Drobotenko M., Svidlov A., Gerasimenko E., Malyshko V., Elkina A., Baryshev M., Dzhimak S. (2020). Inequality in the Frequency of the Open States Occurrence Depends on Single ^2^H/^1^H Replacement in DNA. Molecules.

[66] Coquery M., Villeneuve J.P. (2001). Final Report on the Split Sampling Exercises and Quality Assurance Activities.

[67] Skripnikov A.Y., Polyakov N.B., Tolcheva E.V., Velikodvorskaya V.V., Dolgov S.V., Demina I.A., Rogova M.A., Govorun V.M. (2009). Proteome Analysis of the Moss Physcomitrella patens (Hedw.) B.S.G.A. Biochemistry (Moscow).

[68] O’Farrell P.H. (1975). High resolution two-dimensional electrophoresis of proteins. J. Boil. Chem..

[69] Blum H., Beier H., Gross H.J. (1987). Improved silver staining of plant proteins, RNA and DNA in polyacrylamide gels. Electrophoresis.

[70] Shevchenko A., Tomas H., Havli J., Olsen J.V., Mann M. (2006). In-gel digestion for mass spectrometric characterization of proteins and proteomes. Nat. Protoc..

[71] Savitzky A., Golay M.J.E. (1964). Smoothing and Differentiation of Data by Simplified Least Squares Procedures. Anal. Chem..

[72] Makarova M.P., Syroeshkin A.V., Maksimova T.V., Matveeva I.S., Pleteneva T.V. (2019). Features of the rapid determination of trace elements in medicinal and unofficial plants. Dev. Regist. Med..

[73] Syroeshkin A., Uspenskaya E., Pleteneva T., Morozova M., Maksimova T., Koldina A., Makarova M., Levitskaya O., Zlatskiy I. (2019). Mechanochemical activation of pharmaceutical substances as a factor for modification of their physical, chemical and biological properties. Int. J. Appl. Pharm..

[74] Kovalenko V.F., Zlatskiy I.A. (2019). Toxicity of chlorophenols in embryogenesis of fishes. Hydrobiol. J..

